# Liver Cancer: Improving Standard Diagnosis and Therapy

**DOI:** 10.3390/cancers15184602

**Published:** 2023-09-17

**Authors:** Ryota Masuzaki

**Affiliations:** Division of Gastroenterology and Hepatology, Department of Medicine, Nihon University School of Medicine, Tokyo 173-8610, Japan; masuzaki.ryota@nihon-u.ac.jp; Tel.: +81-3-3972-8111; Fax: +81-3-3956-8496

In 2020, liver cancer ranked sixth for incidence (841,000 cases) and fourth for deaths globally (782,000 cases) [1], with the most common type of liver cancer being hepatocellular carcinoma (HCC), which represents 75–85% of liver cancer cases. Thus far, significant advances have been made in curative and palliative HCC treatment, and in this Special Issue, we focus on both basic and clinical research for recent advances in HCC diagnosis and treatment. This Special Issue comprises eleven articles (nine original articles and two reviews) presented by outstanding researchers in the field of hepatology. 

Curative treatment involves surgical resection and locoregional therapy (often performed via ultrasound guidance). Ohama et al. compared the initial recurrence of surgical resection and ultrasound-guided radiofrequency ablation (RFA) and found no significant differences in recurrence-free survival in early-stage HCC [2]. The SURF trial compared RFA and surgery for HCC, and both treatments showed comparable recurrence-free survival and overall survival for small HCC [3]. This study also further supported the curative role of locoregional therapy in early HCC.

Zhang et al. analyzed infectious complications after thermal ablation of HCC and their impact on overall survival [4]. They found post-operative infection affected local tumor progression rate (*p* = 0.028) and overall survival (*p* = 0.049). Although the reason remains unclear, tumor size, biliary invasion, and advanced portal hypertension may contribute to this result. Chen et al. reported the clinical features of post-RFA fever. They found the independent factors associated with fever were younger age, low albumin, general anesthesia, tumor size, and tumor number [5]. Huh et al. performed a metanalysis to investigate the accuracy of radiological features in diagnosing tumor viability [6]. Nodular, mass-like, or irregular thick tissue with arterial phase hyperenhancement had the highest sensitivity and diagnostic odds ratio for diagnosing viable HCC. A similar outcome of RFA with surgery for small HCC can only be accomplished by meticulous techniques to achieve complete ablation with proper planning and management of complications [7].

Savic et al. compared the efficacy and safety of conventional and drug-eluting beads (DEB) trans-arterial chemoembolization (TACE) [8]. Although there were no significant differences in overall survival in conventional TACE and DEB-TACE, infiltrate or nodular features affected the overall survival.

Immune checkpoint inhibitors (ICI) have become the standard of care for intermediate and advanced stages of HCC. Sinner et al. reported the treatment efficacy and safety of atezolizumab and bevacizumab as a second or later regimen [9]. This multicenter study found that median overall survival was 16.0 months (95% confidence interval [CI], 5.6–26.4 months), and progression-free survival was 7.1 months (95% CI, 4.4–9.8 months). Recently, a combination of cytotoxic T-lymphocyte-associated antigen 4 (CTLA-4) and programmed cell death ligand 1 (PD-L1) inhibitors, tremelimab and durvalumab, showed better overall survival than sorafenib [10] and was incorporated into unresectable HCC treatment.

Lenvatinib, an oral tyrosine inhibitor (TKI), showed non-inferior survival to sorafenib. Amioka et al. reported the efficacy of lenvatinib for intermediate-stage unresectable HCC [11]. They included 140 patients with TACE-refractory or -unsuitable HCC, and although the study was retrospective, some patients had good responses in TACE after initiation of lenvatinib. Additionally, Amioka et al. also analyzed the association between radiologic response and overall survival when treated with lenvatinib and found that initial radiologic response was an independent prognostic factor for overall survival in multivariate analysis [12].

Kuo et al. analyzed the virologic status of viral HCC patients treated with sorafenib [13]. They first compared the overall survival of hepatitis B virus (HBV)-related HCC and hepatitis C virus (HCV)-related HCC and detected no significant difference. Next, they analyzed the hazard ratio regarding survival and found that well-controlled viremia was associated with better survival in multivariate analysis (hazard ratio, 0.63; 95% CI, 0.42–0.93; *p*-value, 0.022). The treatment option for HCC highly depends on the liver function reservoir, and thus, the treatment of background liver disease should be considered simultaneously. Indeed, medical interventions for background liver disease in non-viral HCC are an unmet need. Furthermore, predictive factors for responders and the management of immune-related adverse events are relevant in daily clinical practice. 

Suppressors of cytokine signaling (SOCS) and HCC were narratively summarized by Masuzaki et al. [14]. SOCS protein negatively regulates cytokine signaling related to cell proliferation, cell growth, insulin signaling, and immune response, and whether it can be a treatment target for HCC or chronic liver disease should be evaluated in future studies. Additionally, Gutiérrez Sáenz de Santa María et al. investigated the role of folic acid in liver regeneration and presented a possible role in patients undergoing hepatectomy [15].

In conclusion, early diagnosis and proper treatments from the early stage to the advanced stage of liver cancer are crucial to improve overall survival. Treatments include antitumor as well as hepatitis treatment to maintain a good liver function reservoir. Lastly, it is clear that ICI has opened a new era for liver cancer treatment, and strategies to predict and improve ICI responses should be investigated in future clinical and basic research.

## Abbreviations

CI, confidence interval; CTLA4, cytotoxic T-lymphocyte-associated antigen 4; DEB, drug-eluting beads; HBV, hepatitis B virus; HCC, hepatocellular carcinoma; HCV hepatitis C virus; ICI, immune checkpoint inhibitor; PD-L1, programmed cell death ligand 1; RFA, radiofrequency ablation; SOCS, suppressor of cytokine signaling; TACE, trans-arterial chemoembolization; TKI, tyrosine kinase inhibitor.
